# Erratum: Why vitamin D for cancer patients?

**DOI:** 10.3332/ecancer.2021.1328

**Published:** 2021-12-02

**Authors:** S Gandini, FP Francesco, H Johanson, B Bonanni, A Testori

**Affiliations:** 1 Epidemiology and Biostatistics Division, European Institute of Oncology, 20141 Milan, Italy; 2 Melanoma and Muscle-Cutaneous Sarcomas Division, European Institute of Oncology, 20141 Milan, Italy; 3 Cancer Prevention and Genetics Division, European Institute of Oncology, 20141 Milan, Italy

FP Francesco is corrected to PF Ferrucci (Pier Francesco Ferrucci).

